# A population-based cohort study of the risk of colorectal and other cancers among users of low-dose aspirin

**DOI:** 10.1038/sj.bjc.6600760

**Published:** 2003-03-04

**Authors:** S Friis, H T Sørensen, J K McLaughlin, S P Johnsen, W J Blot, J H Olsen

**Affiliations:** 1 Institute of Cancer Epidemiology, Danish Cancer Society, DK-2100 Copenhagen, Denmark; 2 Department of Clinical Epidemiology, Aarhus University Hospital and Aalborg Hospital, DK-8600 Aarhus and DK-9100 Aalborg, Denmark; 3 Department of Medicine V, Aarhus University Hospital, DK-8600 Aarhus, Denmark; 4 International Epidemiology Institute, Rockville, Maryland 20850, USA; 5 Department of Medicine, Vanderbilt University Medical Center, Vanderbilt-Ingram Comprehensive Cancer Center, Nashville, TN, USA; 6 Department of Medicine M, Aalborg Hospital, DK-9100 Aalborg, Denmark

**Keywords:** cancer, low-dose aspirin, risk, epidemiology, cohort study

## Abstract

Using data from the population-based Prescription Database of North Jutland County and the Danish Cancer Registry, we compared cancer incidence among 29 470 individuals prescribed low-dose aspirin at maximum doses of 150 mg with expected incidence based on county-specific cancer rates, during a 9-year study period. We observed 2381 cancer cases compared with 2187 expected, yielding a standardised incidence ratio (SIR) of 1.09 (95% confidence interval (CI), 1.05–1.13). No apparent risk reductions were found for cancers of the colon (SIR, 0.9; 95% CI, 0.7–1.1) or rectum (SIR, 1.0; 95% CI, 0.8–1.2), or for other site-specific cancers. Increased SIRs were observed for kidney cancer (SIR, 1.4; 95% CI, 1.1–1.7) and brain cancer (SIR, 1.7; 95% CI, 1.3–2.2), although the excess in the latter was confined to the first year of follow-up. Stratification by number of prescriptions and duration of follow-up revealed no apparent trends. The SIR for colorectal cancer was close to unity (SIR, 0.9; 95% CI, 0.6–1.2) among persons with 10 or more prescriptions who were followed for at least 5 years. Our results do not support a major protective effect of low-dose aspirin on the development of colorectal or other cancers. The observed excesses of kidney and brain cancers are not likely to be causally related to the use of low-dose aspirin.

Epidemiologic and experimental evidence strongly suggests that use of aspirin and other nonsteroidal anti-inflammatory drugs (NSAIDs) reduces the risk for colorectal cancer (Thun *et al*, 2002). A recent epidemiologic review (Garcia-Rodriguez and Huerta-Alvarez, 2001) reported pooled relative risk estimates for colorectal cancer of 0.71 (95% CI, 0.65–0.76) for use of aspirin and 0.63 (95% CI, 0.57–0.70) for use of nonaspirin NSAIDs. The extent to which aspirin and other NSAIDs protect against cancers other than colorectal cancer has not been established. There is some epidemiologic evidence that use of aspirin and other NSAIDs reduces the risk for cancers of the stomach and oesophagus (Thun *et al*, 1993; Farrow *et al*, 1998; Coogan *et al*, 2000), whereas the results are conflicting for nongastrointestinal cancers, including cancers of the lung (Thun *et al*, 1993; Schreinemachers and Everson, 1994; Langman *et al*, 2000), breast (Khuder and Mutgi, 2001; Cotterchio *et al*, 2001; Meier *et al*, 2002), prostate (Norrish *et al*, 1998; Langman *et al*, 2000; Leitzmann *et al*, 2002), bladder (Castelao *et al*, 2000; Langman *et al*, 2000), and ovary (Akhmedkhanov *et al*, 2001; Meier *et al*, 2002).

Only limited data are available on what dose and duration of treatment with aspirin and other NSAIDs are necessary to reduce the risks for colorectal and, potentially, other cancers. Some studies have evaluated the association between regular use of low-dose aspirin and colorectal cancer risk (Giovannucci *et al*, 1995; Rosenberg *et al*, 1998; Stürmer *et al*, 1998; Garcia-Rodriguez and Huerta-Alvarez, 2001), but the results are conflicting and little information has been provided for other cancer sites. Any substantial cancer-preventive effect of low-dose aspirin would have important public health implications. We have therefore examined the incidence of colorectal and other cancers in a Danish population-based cohort of persons receiving prescriptions for low-dose aspirin.

## MATERIAL AND METHODS

The study was conducted within the population of North Jutland, a county with nearly 500 000 inhabitants, representing approximately 9% of the total Danish population. Through a computerised accounting system maintained by Danish pharmacies, the tax-supported health insurance programme in Denmark refunds part of the costs of most drugs, including some over-the-counter medications, if prescribed by physicians. In North Jutland, this accounting system also provides prescription data to the Pharmacoepidemiologic Prescription Database (Gaist *et al*, 1997), initiated in 1989, which retains key data on prescriptions for refundable drugs dispensed from all pharmacies in the county. The data includes the type of drug prescribed according to the Anatomical Therapeutical Chemical (ATC) classification system (Capellá, 1993), tablet size, date of prescription, and the patient's civil registry number (a unique number assigned to all Danish residents that encodes gender and date of birth). The use of the civil registry number ensures that complete individual prescription histories can be established, and permits valid linkage of information between registers.

Through the Prescription Database, we identified 32 794 individuals prescribed low-dose aspirin between 1 January 1989 and 31 December 1995. These prescriptions were identified by the ATC codes for aspirin (B01AC06 and N02BA01) in tablet sizes of 75, 100 or 150 mg. All preparations of aspirin are available over-the-counter in Denmark, but low-dose aspirin, which is used almost exclusively for secondary prevention of cardiovascular disease, is generally prescribed by physicians, being reimbursable by 50% through the national health insurance programme. In Denmark, the recommended dose of low-dose aspirin for secondary prevention of cardiovascular disease is 75–150 mg once daily, and the preparation is mainly prescribed in packets for 3-month use (Sørensen *et al*, 2000).

Overall, 668 (2.0%) of the identified persons prescribed low-dose aspirin were excluded because of (i) residency outside the county of North Jutland at the date of prescription (*n*=617); (ii) an invalid civil registry number (*n*=15); (iii) death prior to or at the date of prescription (*n*=19); or (iv) parent (of patient) registered as customer (*n*=17). After these exclusions, 32 126 (98.0%) persons were left for subsequent record linkage.

Information on cancer occurrence was obtained by linkage to the Danish Cancer Registry, which has recorded incident cases of cancer on a nationwide basis since 1943 (Storm *et al*, 1997). Cancers were classified according to a modified Danish version of the International Classification of Diseases, 7th revision (Storm *et al*, 1997). Study subjects with a cancer diagnosis, except nonmelanoma skin cancer, prior to the date of first recorded prescription for low-dose aspirin (*n*=2656; 8.1%) were excluded, leaving a final study cohort of 29 470 (89.9%) individuals.

The observation period for cancer began on the date of the first recorded prescription for low-dose aspirin and ended on the date of first primary cancer diagnosis except nonmelanoma skin cancer (*n*=1962), date of death (*n*=8493), or 31 December 1997 (*n*=19 015), whichever occurred first. Information on date of death was obtained through linkage to the National Mortality Files (Juel and Helweg-Larsen, 1999). The observed number of cancer cases among the cohort members was compared with the number expected based on cancer incidence rates among the general population of North Jutland. To obtain expected numbers, county-specific incidence rates of first primary cancers, calculated by sex, 5-year age groups, and 5-year calendar periods, were applied to the corresponding person-years of the cohort members. The standardised incidence ratio (SIR), that is, the ratio of the observed to the expected number of cancer cases, and 95% confidence intervals (CIs) were calculated for total cancer and for specific sites based on the assumption that the observed cancer cases followed a Poisson distribution (Bailar and Ederer, 1964). We also computed SIRs stratified by the number of low-dose aspirin prescriptions and by years of follow-up. For these analyses, each study subject contributed person-years to one to four categories of prescription frequency (1; 2–4; 5–9; or ⩾10 prescriptions), with follow-up for cancer (<1; 1–4; 5–9 years) beginning on the date of the first prescription within the given category of prescription frequency. A test for linear trend was used to evaluate trends in SIRs with the number of low-dose aspirin prescriptions or years of follow-up.

## RESULTS

Characteristics of the study cohort of 15 058 men (51%) and 14 412 women (49%) are presented in Table 1

**Table 1:** Characteristics of users of low-dose aspirin recorded in the Prescription Database of North Jutland County, Denmark, between 1989 and 1995

**Characteristic**	**No.**	**(%)**
Total no. of patients	29 470	100
Men	15 058	51
Women	14 412	49
		
Age at entry^a^ (years)		
<50	1751	6
50–69	11 456	39
⩾70	16 263	55
		
Mean age at entry^a^	70 years	
		
Year of entry^a^		
1989–1991	11 044	37
1992–1993	9000	31
1994–1995	9426	32
		
Number of prescriptions		
1	5794	20
2–4	7069	24
5–9	7091	24
⩾10	9516	32

a Date of first recorded prescription for low-dose aspirin.

. The mean age at cohort entry, that is, time of first recorded prescription for low-dose aspirin, was 70 years, with only 6% of the cohort being younger than 50 years at entry. The cohort members accrued a total of 121 562 person-years, with an average length of follow-up since the first recorded prescription for low-dose aspirin of 4.1 years (range, 0–9 years); 80% of subjects received two or more prescriptions for low-dose aspirin, and 32% received 10 or more prescriptions.

Overall, we observed 2381 cancer cases compared with 2187 expected, yielding a SIR of 1.09 (95% CI, 1.05–1.13) (Table 2

**Table 2:** Standardised incidence ratios (SIR) and 95% confidence intervals (CI) for cancers at selected sites, stratified by sex,^a^ among users of low-dose aspirin in North Jutland county, Denmark, 1989–1997

	**Men**			**Women**			**Total**		
**Site of cancer (modified ICD-7 code)**	**Obs^b^**	**SIR**	**95% CI**	**Obs^b^**	**SIR**	**95% CI**	**Obs^b^**	**SIR**	**95% CI**
All malignant neoplasms (140–205)	1362	1.10	1.04–1.16	1019	1.08	1.01–1.14	2381	1.09	1.05–1.13
Buccal cavity and Pharynx (140–148)	32	1.2	0.8–1.7	16	1.5	0.9–2.4	48	1.3	0.9–1.7
Digestive system (150–159)	303	1.0	0.9–1.1	247	1.0	0.9–1.2	550	1.0	0.9–1.1
Oesophagus (150)	17	1.3	0.7–2.0	9	1.5	0.7–2.8	26	1.3	0.9–1.9
Stomach (151)	50	1.1	0.8–1.5	18	0.9	0.5–1.3	68	1.1	0.8–1.3
Colon (153)	108	1.1	0.9–1.3	87	0.9	0.7–1.1	195	0.9	0.7–1.1
Rectum (154)	67	1.0	0.8–1.3	37	0.9	0.6–1.3	104	1.0	0.8–1.2
Liver (155)	16	1.1	0.6–1.7	5	0.7	0.2–1.7	21	1.0	0.6–1.5
Pancreas (157)	27	0.9	0.6–1.4	35	1.2	0.8–1.6	62	1.1	0.8–1.3
Larynx (161)	16	1.4	0.8–2.3	4	2.7	0.7–6.8	20	1.5	0.9–2.4
Lung (162)	173	1.0	0.8–1.1	101	1.3	1.0–1.5	274	1.1	0.9–1.2
Breast (170)	1	0.6	0.0–3.2	148	0.9	0.8–1.1	149	0.9	0.8–1.1
Female genital organs (171–176)	—	—	—	108	1.1	0.9–1.3	108	1.1	0.9–1.3
Cervix uteri	—	—	—	15	0.9	0.5–1.6	15	0.9	0.5–1.6
Corpus uteri	—	—	—	45	1.1	0.8–1.5	45	1.1	0.8–1.5
Ovary	—	—	—	34	1.1	0.7–1.5	34	1.1	0.7–1.5
Prostate (177)	196	1.1	1.0–1.3	—	—	—	196	1.1	1.0–1.3
Kidney (180)	45	1.6	1.1–2.1	22	1.1	0.7–1.6	67	1.4	1.1–1.7
Urinary bladder (181)	134	1.2	1.0–1.5	27	0.9	0.6–1.4	161	1.2	1.0–1.4
Melanoma (190)	31	1.3	0.9–1.8	21	1.1	0.7–1.7	52	1.2	0.9–1.6
Nonmelanoma skin cancer (191)	246	1.1	0.9–1.2	161	1.0	0.9–1.2	407	1.1	0.9–1.2
Brain (193)	33	1.5	1.0–2.1	37	1.9	1.4–2.7	70	1.7	1.3–2.2
Non-Hodgkin's lymphoma (200, 202)	37	1.4	1.0–1.9	20	0.9	0.5–1.4	57	1.1	0.9–1.5
Leukaemia (204)	44	1.2	0.9–1.7	25	1.4	0.9–2.0	69	1.3	1.0–1.6

a Person-years of observation in each stratum: men, 61 093; women, 60 469.

b Observed number of cases.

). No apparent risk reductions were observed for cancers of the gastrointestinal tract, including cancers of the colon (SIR, 0.9; 95% CI, 0.7–1.1), rectum (SIR, 1.0; 95% CI, 0.8–1.2), and stomach (SIR, 1.1; 95% CI, 0.8–1.3). Increased SIRs were found for cancers of the kidney (SIR, 1.4; 95% CI, 1.1–1.7) and brain (SIR, 1.7; 95% CI, 1.3–2.2). Slight risk elevations were seen for several smoking-related cancers, including cancers of the buccal cavity and pharynx (SIR, 1.3; 95% CI, 0.9–1.7), urinary bladder (SIR, 1.2; 95% CI, 1.0–1.4), oesophagus (SIR, 1.3; 95% CI, 0.9–1.9), larynx (SIR, 1.5; 95% CI, 0.9–2.4), and leukaemia (SIR, 1.3; 95% CI, 1.0–1.6), whereas the SIR for lung cancer was close to expectation (SIR, 1.1; 95% CI, 0.9–1.2). The SIR estimates for site-specific cancers were similar in men and women, except for cancer of the kidney (SIR, 1.6 (95% CI, 1.1–2.1) in men *vs* 1.1 (95% CI, 0.7–1.6) in women) and non-Hodgkin's lymphoma (SIR, 1.4 (95% CI, 1.0–1.9) in men *vs* 0.9 (95% CI, 0.5–1.4) in women).

The SIRs did not vary markedly when stratified by age. The SIR for total cancer was 1.14 (95% CI, 1.07–1.22) among persons below 70 years at cohort entry and 1.06 (95% CI, 1.01–1.11) among older persons (data not shown). Similarly, for specific cancer sites, including colorectal cancer and kidney cancer, the SIR estimates did not differ substantially between these two age groups.

Table 3

**Table 3:** Standardised incidence ratios (SIR) and 95% confidence intervals (CI) for selected cancer sites among users of low-dose aspirin, stratified by number of prescriptions and years of follow-up, North Jutland county, Denmark, 1989–1997

	**Follow-up (years)**											
**Site of cancera and**	**<1**			**1–4**			**5–9**			**Total**		
**No. of prescriptions (1,2–4, 5–9, ⩾10)**	**Obs**	**SIR**	**95% CI**	**Obs**	**SIR**	**95% CI**	**Obs**	**SIR**	**95% CI**	**Obs**	**SIR**	**95% CI**
All sites												
1	277	1.3	1.1–1.4	189	1.1	0.9–1.2	59	1.2	0.9–1.5	525	1.2	1.1–1.3
2–4	265	1.1	0.9–1.2	323	1.2	1.0–1.3	60	1.1	0.8–1.4	648	1.1	1.0–1.2
5–9	26	1.3	0.8–1.8	450	1.0	0.9–1.1	71	1.0	0.8–1.2	547	1.0	0.9–1.1
⩾10	1	4.3	0.1–23.7	269	1.1	1.0–1.2	391	1.1	1.0–1.2	661	1.1	1.0–1.2
Total	569	1.2	1.1–1.3	1231	1.1	1.0–1.1	581	1.1	1.0–1.2	2381	1.1	1.0–1.1
Colorectal												
1	27	0.9	0.6–1.3	31	1.3	0.9–1.8	10	1.5	0.7–2.7	68	1.1	0.9–1.4
2–4	39	1.1	0.8–1.6	48	1.3	0.9–1.7	3	0.4	0.1–1.2	90	1.1	0.9–1.4
5–9	3	1.1	0.2–3.1	54	0.9	0.6–1.1	8	0.8	0.3–1.6	65	0.9	0.7–1.1
⩾10	0	—	—	33	0.9	0.7–1.4	43	0.9	0.6–1.2	76	0.9	0.7–1.1
Total	69	1.0	0.8–1.3	166	1.0	0.9–1.2	64	0.9	0.7–1.1	299	1.0	0.9–1.1
Stomach												
1	8	1.2	0.5–2.3	10	1.9	0.9–3.6	1	0.7	0.0–4.0	19	1.4	0.9–2.2
2–4	6	0.8	0.3–1.7	10	1.2	0.6–2.2	2	1.3	0.1–4.6	18	1.0	0.6–1.6
5–9	0	—	—	14	1.0	0.6–1.7	1	0.5	0.0–2.7	15	0.9	0.5–1.5
⩾10	0	—	—	8	1.1	0.5–2.2	8	0.8	0.3–1.5	16	0.9	0.5–1.5
Total	14	0.9	0.5–1.6	42	1.2	0.9–1.6	12	0.8	0.4–1.4	68	1.1	0.8–1.3
Oesophagus												
1	5	2.5	0.8–5.9	2	1.3	0.1–4.5	2	4.6	0.5–16.6	9	2.3	1.0–4.3
2–4	1	0.5	0.0–2.5	2	0.8	0.1–2.8	1	2.0	0.0–11.3	4	0.8	0.2–1.9
5–9	0	—	—	3	0.7	0.1–2.1	2	3.1	0.3–11.1	5	1.0	0.3–2.4
⩾10	0	—	—	4	1.8	0.4–4.6	4	1.2	0.3–3.2	8	1.5	0.6–2.9
Total	6	1.4	0.5–3.0	11	1.1	0.5–1.9	9	1.9	0.9–3.6	26	1.3	0.9–1.9
Kidney												
1	7	1.4	0.5–2.8	4	1.0	0.3–2.5	3	2.7	0.5–7.9	14	1.4	0.7–2.3
2–4	10	1.7	0.8–3.2	2	0.3	0.0–1.1	2	1.7	0.2–6.0	14	1.1	0.6–1.8
5–9	0	—	—	15	1.5	0.8–2.4	2	1.3	0.1–4.5	17	1.4	0.8–2.2
⩾10	0	—	—	9	1.6	0.7–3.1	13	1.7	0.9–2.9	22	1.7	1.0–2.5
Total	17	1.5	0.9–2.4	30	1.1	0.8–1.6	20	1.7	1.1–2.7	67	1.4	1.1–1.7

presents SIR estimates, stratified by number of prescriptions for low-dose aspirin and years of follow-up, for total cancer, selected gastrointestinal cancers, and kidney cancer. Overall, stratification by duration of follow-up yielded SIRs for total cancer of 1.2 (95% CI, 1.1–1.3) for less than 1 year of follow-up, 1.1 (95% CI, 1.0–1.1) for 1–4 years of follow-up, and 1.1 (95% CI, 1.0–1.2) for 5–9 years of follow-up. Similar SIRs were observed for total cancer in the four categories of prescription frequency. The SIRs for colorectal and stomach cancer decreased slightly with both increasing number of prescriptions and increasing years of follow-up, but none of the trends was statistically significant. No consistent trends in risk estimates were observed for cancers of the oesophagus or kidney. Among persons with 10 or more prescriptions, the SIR for kidney cancer was of the same magnitude in the 1- to 4-year (SIR, 1.6; 95% CI, 0.7–3.1) and 5- to 9-year (SIR, 1.7; 95% CI, 0.9–2.9) follow-up periods.

The overall increased SIR for brain cancer was entirely due to an excess in the first year of follow-up (SIR, 4.7; 95% CI, 3.4–6.3) among persons receiving one prescription (SIR, 6.2; 95% CI, 4.1–9.1) or 2–4 prescriptions (SIR, 3.5; 95% CI, 2.0–5.7) (data not shown). For the remaining sites of interest, including cancers of the lung, breast, prostate, bladder, and ovary, stratification by number of prescriptions and years of follow-up revealed no consistent trends (data not shown).

## DISCUSSION

In this population-based cohort study, we found little evidence of a reduced risk for colorectal or other cancers among nearly 30 000 individuals receiving prescriptions for low-dose aspirin. There was a slight decreasing trend in risks for colorectal and stomach cancers with increasing number of prescriptions and length of follow-up, however, the SIRs for both cancers were close to unity among persons with 10 or more prescriptions (equivalent to an estimated period of aspirin therapy of minimum 30 months) who were followed for at least 5 years.

Our results are consistent with those of two previous studies reporting on colorectal cancer risk among users of low-dose aspirin. In the only randomised clinical trial of aspirin and cancer risk published to date (Stürmer *et al*, 1998), based on the Physicians' Health Study in the United States, both randomised (5 years of follow-up) and post-trial observational analyses (up to 12 years of follow-up) revealed no association between the assigned low dose of aspirin (325 mg every other day) and risk for colorectal cancer. Limitations however, include possible exposure misclassification in the post-trial analysis (exposure was assessed only at the beginning of the observational follow-up period) and limited statistical power. A large nested case – control study (Garcia-Rodriguez and Huerta-Alvarez, 2001), based on the General Practice Research Database in the UK, reported a significant reduction in risk for colorectal cancer (OR, 0.6; 95% CI, 0.4–0.9) associated with the current use of aspirin at daily doses of 300 mg or greater for at least 6 months, whereas daily doses of 75 and 150 mg aspirin were not associated with a reduced risk. A limitation of that study was the relatively small number of subjects with long continuous (>2 years) exposure to aspirin. In contrast, a population-based case – control study (Rosenberg *et al*, 1998) and a cohort analysis based on the Nurses' Health Study (Giovannucci *et al*, 1995) reported that regular users of low-dose aspirin had a substantially reduced risk for colorectal cancer. However, the doses examined in the latter study had upper limits intermediate between cardioprotective and anti-inflammatory doses, and no results were presented separately for low-dose aspirin as defined in the present study.

The strengths of our study are the population-based design, the continuously updated data on aspirin use based on a prescription database covering all pharmacies in a Danish county, the relatively large number of outcomes, and the complete follow-up obtained by use of the unique civil registry number and computerised linkage to the Danish Cancer Registry. The Cancer Registry covers the entire population of Denmark and has been shown to have accurate and virtually complete ascertainment of cancer cases (Storm *et al*, 1997). Although low-dose aspirin is available over the counter, a 50% government refund at the time of prescription exists and is used predominantly for conditions requiring physician attendance; thus, we have probably identified most of the patients using low-dose aspirin on a regular basis in the North Jutland population during the period of registration.

In our register-based approach, we had to rely on prescription data, with no information on indications, dose schedules, or degree of compliance. However, a large proportion of our cohort members can be assumed to have received low-dose aspirin for secondary prevention of cardiovascular disease at daily doses of 75–150 mg. Also, the fact that drug exposure was based on prescriptions actually dispensed at pharmacies and paid for in part by the patient implies high compliance. Nonetheless, some misclassification because of noncompliance is possible, leading to attenuation of the risk estimates.

Our study had a number of other limitations. We had no data on over-the-counter purchase of high-dose aspirin. Use patterns of high-dose aspirin and nonaspirin NSAIDs for analgesic or anti-inflammatory purposes may have been different among persons prescribed low-dose aspirin than among the general population, thus introducing possible exposure misclassification of aspirin and/or confounding by nonaspirin NSAIDs. The finding of slightly increased SIRs for several smoking-related cancers is consistent with the fact that smoking is a strong independent risk factor for both ischaemic cardiovascular disease and cancer (Dreyer and Olsen, 1998), and that low-dose aspirin in Denmark is prescribed almost exclusively as secondary prevention in patients with cardiovascular disease. Our inability to adjust for concurrent NSAID use, smoking, and other potential confounders may have obscured a cancer-preventive effect of low-dose aspirin. Nevertheless, the absence of any substantial trends in SIRs with the number of prescriptions of low-dose aspirin or years of follow-up argues against any important inverse association between use of low-dose aspirin and cancer risk.

The increased risk for kidney cancer may partly reflect an increased prevalence of hypertension among the study subjects with ischaemic heart disease. Patients with kidney tumours may present with hypertension some years prior to kidney cancer diagnosis (Steffens *et al*, 1992), and there is increasing evidence that hypertension *per se* increases the risk for kidney cancer (McLaughlin and Lipworth, 2000). Other arguments against a causal relation between the use of low-dose aspirin and kidney cancer are the gender difference and the absence of a dose – response relation or temporal trend in the risk estimates for kidney cancer in this study, in addition to the largely null results from previous epidemiological studies of aspirin intake and kidney cancer (McLaughlin and Lipworth, 2000).

The increased risk observed for brain cancer is likely to be a result of ‘reverse causation’ (i.e. prescription for low-dose aspirin to treat symptoms of brain cancer). The excess risk was confined exclusively to the first year of follow-up among patients who received only a few prescriptions for low-dose aspirin. Some patients with brain tumours may have received anti-thrombotic treatment with low-dose aspirin prior to diagnosis, because they presented with symptoms resembling thrombotic cerebral diseases, for example, transient ischaemic attacks.

The duration of exposure to aspirin necessary to prevent colorectal or other cancers has not been firmly established. In one study, a protective effect at doses of at least 300 mg daily became evident after only 6 months of continuous treatment (Garcia-Rodriguez and Huerta-Alvarez, 2001), but other studies have suggested that longer periods of treatment are necessary to achieve a protective effect. Our observation period of up to 9 years with an average follow-up of 4.1 years may be too short to detect a cancer-preventive effect, even if the effect is on disease progression. However, a substantial proportion of the patients were likely to be prevalent users at the start of the registration period and therefore to have had exposure before the observation period.

In summary, patients prescribed low-dose aspirin at doses of maximum 150 mg were not at a substantially reduced risk for colorectal or other cancers. The available evidence so far does not support a major protective effect of low-dose aspirin on the development of colorectal or other cancers. Further studies are needed to establish what dose and duration of treatment by aspirin and other NSAIDs are necessary to prevent colorectal and possibly other cancers.
